# Involvement of the pulmonary arteries in patients with Takayasu arteritis: a prospective study from a single centre in China

**DOI:** 10.1186/s13075-020-02203-1

**Published:** 2020-06-05

**Authors:** Xiufang Kong, Lili Ma, Peng Lv, Xiaomeng Cui, Rongyi Chen, Zongfei Ji, Huiyong Chen, Jiang Lin, Lindi Jiang

**Affiliations:** 1 Department of Rheumatology, Zhongshan Hospital Affiliated to Fudan University, Shanghai, China; 2 Department of Radiology, Zhongshan Hospital Affiliated to Fudan University, Shanghai, China; 3 Center of Clinical Epidemiology and Evidence-based Medicine, Fudan University, Shanghai, China

**Keywords:** Takayasu arteritis, Pulmonary artery involvement, Cardiac function, Pulmonary lesions

## Abstract

**Background:**

Takayasu arteritis (TA) is a large vessel vasculitis that can involve pulmonary arteries (PAs). We studied multiple clinical characteristics related to pulmonary artery involvement (PAI) in TA patients.

**Methods:**

We enrolled 216 patients with TA from a large prospective cohort. PAI was assessed in each patient based on data from magnetic resonance angiography/computed tomography angiography. Pulmonary hypertension, cardiac function, and pulmonary parenchymal lesions were evaluated further in patients with PAI based on echocardiography, the New York Heart Association Functional Classification, and pulmonary computed tomography, respectively. These abnormalities related to PAI were followed up to evaluate treatment effects.

**Results:**

PAI was detected in 56/216 (25.93%) patients, which involved the pulmonary trunk, main PAs, and small vessels in the lungs. Among patients with PAI, 28 (50%) patients were accompanied by pulmonary hypertension, which was graded as ‘severe’ in 9 (16.07%), ‘moderate’ in 10 (17.86%), and mild in 9 (16.07%). Twenty-six (46.43%) patients showed advanced NYHA function (III, 20, 35.71%; IV, 6, 10.71%). Furthermore, 21 (37.50%) patients presented with abnormal pulmonary parenchymal lesions in the area corresponding to PAI (e.g. the mosaic sign, infarction, bronchiectasis). During follow-up, two patients died due to heart failure and pulmonary thrombosis. In the remaining patients, the abnormalities mentioned above improved partially after routine treatment.

**Conclusions:**

PAI is common in TA patients. PAI can cause pulmonary hypertension, cardiac insufficiency, and pulmonary parenchymal lesions, which worsen patients’ prognosis.

## Introduction

Takayasu arteritis (TA) is a chronic, granulomatous large vessel vasculitis. It involves the aorta and its main branches predominantly and leads to vascular thickness, stenosis, and occlusion [1]. In addition, TA occurs preferentially in young (20–40 years) women and has a relatively high prevalence in Asian countries compared with that in the USA and European countries [2].

Besides the aorta and its branches, the pulmonary arteries (PAs) are involved in TA. PAs have been reported to be involved in 6.9 to 80% of TA patients from different populations [3–8]. The stenosis, occlusion, or embolism of PAs can cause pulmonary hypertension (PH), perfusion defects, or even pulmonary infarction [9–11]. Recently, we also found multiple other pulmonary parenchymal lesions in the lung lobes with pulmonary artery involvement (PAI). In addition, right ventricular function is susceptible to damage in patients with PH due to increased afterload, which has a direct adverse impact on the prognosis [12]. Therefore, PAI in patients with TA is life-threatening because it damages the cardiopulmonary function.

Multiple imaging modalities can be employed to detect PA abnormalities. These include digital subtraction angiography (DSA), magnetic resonance angiography (MRA), computed tomography angiography (CTA), positron emission tomography-computed tomography (PET-ECT), and lung ventilation/perfusion (VQ) scan [10, 13, 14]. Among them, DSA is applied rarely due to its invasiveness although it is the ‘gold standard’ to assess PAI. By contrast, CTA and MRA are undertaken more frequently because they show the structure of and inflammation in the blood vessels, respectively [15]. PET-CT can also be employed to detect vascular inflammation [16]. Lung VQ scans can display the vascular emboli for segmental (or sub-segmental) PAs [10].

We investigated the clinical characteristics, pulmonary parenchymal lesions, and cardiac functions in TA patients with PAI by combining multiple imaging modalities (MRA, CTA, PET-CT, lung VQ scan, echocardiography, and high-resolution computed tomography (HRCT). Our aim was to elicit a better understanding of TA patients with PAI to aid rational treatment for these patients and improve their prognosis.

## Methods and materials

### Patients

This was an observational study based on a prospective cohort named East China Takayasu Arteritis (clinical trial number, NCT03893136). This cohort was established from a single centre (Zhongshan Hospital, Fudan University) in China, and all the patients in this cohort were Han Chinese. In this cohort, all patients were classified as TA according to the classification criteria set by the American College of Rheumatology in 1990 [17]. The clinical data of this cohort were recorded using a standardised form and inputted into an electronic database. Patients diagnosed from 1 January 2013 to 31 May 2019 were enrolled in the present study. Patients with concurrent disorders that could involve the lungs (e.g. pulmonary infection, asthma, tumours) were excluded. Finally, 216 patients were included in this study (flowchart was shown as Supplementary Figure 1).

### Disease assessment

At the initial visit, demographic data and clinical features (systemic and ischaemic symptoms, physical signs) were recorded. Laboratory workup (routine tests for blood and urine, erythrocyte sedimentation rate (ESR), C-reactive protein (CRP) level, liver function test, kidney function test, hepatitis-B/C test, interferon-gamma release assay, antibodies against *Mycoplasma* species, sputum pathogens) was undertaken.

Vascular involvements were evaluated by whole-body MRA. If MRA was contraindicated, CTA or PET-CT of the thoraco-abdominal region was done. The type of vascular involvement in TA patients was classified according to the imaging classification created by Hata et al. [18]. Echocardiography was also carried out to evaluate patients' cardiopulmonary conditions.

Pulmonary CT was undertaken for each patient. Additional pulmonary CTA or lung VQ scans were carried out for patients in whom a pulmonary embolism/thrombosis was suspected. CT-guided transthoracic lung biopsy was conducted for patients with confusing pulmonary lesions. Histopathology studies using staining (haematoxylin and eosin, immunohistochemical, acid-fast, silver) were done to evaluate vascular inflammation and detect possible pulmonary infections. Tissue culture was also carried out to clarify if the pulmonary lesions were caused by an infection.

The Kerr criteria [19] were used to assess disease activity: (i) systemic symptoms (infection, tumours, etc. were excluded), (ii) elevated ESR level, (iii) symptoms or signs of vascular ischaemia (weakened pulse or pulselessness, vascular bruits, asymmetric blood pressure), and (iv) positive imaging results. New onset or worsening of two or more criteria indicated ‘active disease’.

### Follow-up

Patients were followed up each month during the first 6 months and every 2–3 months after that as planned. During follow-up, MRA was repeated every 6 months, whereas other imaging examinations (e.g. pulmonary HRCT, echocardiography) were employed as clinically required.

### Treatment

The therapeutic procedure was divided into induction treatment and maintenance treatment. During the induction phase, prednisone (0.8–1.0 mg/kg/day, p.o.) was administered. After 4 weeks, the prednisone dose was tapered gradually to a maintenance dose of 0.1–0.2 mg/kg/day within the next 5 months. Meanwhile, an immunosuppressant (cyclophosphamide (CYC; 0.6–0.8 g/month, i.v.), methotrexate (MTX; 10–15 mg/week, p.o.), azathioprine (AZA; 50–100 mg/day, p.o.), leflunomide (LEF; 10–20 mg/day, p.o.)) or a biological agent was used according to the discretion of the treating physician.

In the maintenance phase, MTX (10–15 mg/week, p.o.), AZA (25–50 mg/day, p.o.), or LEF (10–20 mg/day, p.o.) was administrated.

For patients suspected of having pulmonary thrombosis, anticoagulant therapy (e.g. warfarin) was given. For patients with PH, bosentan, sildenafil, or Adempas was also prescribed according to clinical conditions.

### Pulmonary arterial involvement

For newly diagnosed patients with TA, their whole-body MRA, echocardiology, and pulmonary CT were routinely performed. Combining patients’ symptoms and imaging results, patients’ PAI can be preliminarily evaluated, then patients with suspicious pulmonary lesions will be required to perform extra examinations such as pulmonary CTA or lung VQ scan to confirm it.

Vascular inflammation, as well as stenosis, dilation, or occlusion of the pulmonary trunk (PT), right/left main PAs, or branches of the right/left main PAs, were identified upon MRA or CTA. Thrombosis/embolism in sub-segmental PAs was confirmed by lung VQ scans combined with CTA. ‘Vascular inflammation’ was defined as an increased signal intensity of the vascular wall upon MRA compared with that of the back muscle situated beside the vertebral column of the same slice [15] or standard uptake value (SUV) of ^18^F-fluorodeoxyglucose on PET-CT > 2.5 in the PA [19]. Vascular stenosis was assessed if the vascular diameter of the lesioned segment was less than that of the adjacent normal segment. Vascular dilation was diagnosed if the vascular diameter of the lesioned segment was < 50% greater than the vascular diameter of the upper or lower normal segments. A ‘vascular aneurysm’ was documented if the vascular diameter of the lesioned segment was > 50% of the vascular diameter of the upper or lower normal segments.

Imaging results were analysed independently by two experienced radiologists blinded to the clinical data. Discordant interpretations were settled by a discussion based on additional clinical information until consensus was reached.

### PH and heart function

PH was considered if patients had typical clinical symptoms (dyspnoea, chest pain, haemoptysis) and satisfied at least one of the following criteria: (i) mean PA pressure ≥ 25 mm, pulmonary artery wedge pressure ≤ 15 mmHg, and pulmonary vascular resistance > 3 Wood units at rest as assessed by right heart catheterization (RHC) [20] and (ii) estimated pulmonary arterial systolic pressure (ePASP) ≥ 40 mmHg and peak systolic velocity of the tricuspid valve > 3.4 m/s upon echocardiography [21]. Echocardiography was applied in patients with RHC contraindications. PH classes were also analysed in TA patients according to different causes: left heart disease, lung disease and/or hypoxia, chronic thromboembolism, or unknown multifactorial mechanisms [22].

Given the diagnosis of PH, its severity should be evaluated further. Assessments such as the 6-min walk test and measurement of biomarkers (e.g. brain natriuretic peptide, cardiac troponin T) were undertaken. According to the ePASP upon echocardiography, patients were classified as ‘mild’ (40–54 mmHg), ‘moderate’ (55–64 mmHg), or ‘severe’ (> 65 mmHg) [23].

Impaired right heart function occurs frequently in patients with chronic pulmonary disorders (especially PH). Cardiac function was also assessed in patients with PAI according to the New York Heart Association (NYHA) Functional Classification [24].

### Pulmonary parenchymal lesions

Pulmonary parenchymal lesions within lung segments with PAI were analysed by pulmonary CT or PET-CT. Typical pulmonary features due to PA abnormalities (the mosaic sign, pleural effusion, bronchiectasis, pulmonary infarction) were analysed.

### Statistical analyses

Categorical variables are presented as frequencies and percentages. Continuous variables are presented as the mean ± SD. The significance of those parameters among the two groups was determined by the chi-squared test, Fisher’s exact test, or unpaired Student’s *t* test, as appropriate. *P* < 0.05 (two-sided) was deemed significant. SPSS v20.0 (IBM, Armonk, NY, USA) was used for statistical analyses.

## Results

### Characteristics of the study cohort

The demographic and clinical features of patients are listed in Table 1. Among the 216 patients, 118 (54.63%) patients had ‘active’ disease status according to the Kerr score. The most frequent imaging type was type V (94, 43.52%), followed by type I (61, 28.24%). The pulmonary symptoms of chest pain/distress, cough/sputum, and haemoptysis were present in 51/216 (23.6%), 9/216 (4.17%), and 5/216 (2.31%) patients, respectively. PAI was detected in 56/216 (25.93%) patients. Moreover, 34/216 (15.74%) patients were assessed as having PH.

**Table 1:** Demographic and clinical features

Parameter	Total (*n* = 216)	Patients without PAI (*n* = 160)	Patients with PAI (*n* = 56)	P*
General information				
Female:male	178:37	130:30	48:7	0.41
Age at diagnosis (years, mean ± SD)	35.19 ± 14.75	34.23 ± 15.05	37.96 ± 13.57	0.11
Active status (*n* (%))	118 (54.63)	91 (56.88)	27 (48.21)	0.63
Observational period (month, quartile)	12 (7, 25)	12 (4.5, 24)	11 (6.7, 27)	0.43
Clinical symptoms				
Headache/dizziness (*n* (%))	87 (40.28)	73 (45.63)	14 (25.00)	**0.023**
Weakness (*n* (%))	64 (29.63)	53 (33.13)	11 (19.64)	0.16
Chest pain/distress	51 (23.61)	27 (16.88)	24 (42.86)	**< 0.001**
Fever (*n* (%))	26 (12.04)	24 (15.00)	2 (3.57)	**0.048**
Amaurosis (*n* (%))	25 (11.57)	21 (13.13)	4 (7.14)	0.46
Weight loss (*n* (%))	20 (9.26)	17 (10.63)	3 (5.36)	0.42
Oral ulcer (*n* (%))	12 (5.56)	6 (3.75)	6 (10.71)	**0.04**
Cough/sputum (*n* (%))	9 (4.17)	3 (1.88)	6 (10.71)	**0.008**
Haemoptysis (*n* (%))	5 (2.31)	2 (1.25)	3 (5.36)	0.092
Clinical signs				
Vascular murmur (*n* (%))	68 (31.48)	46 (28.75)	22 (39.29)	0.087
Hypertension (*n* (%))	51 (23.61)	35 (21.88)	16 (28.57)	0.19
Pulselessness/weak pulse (*n* (%))	46 (21.30)	32 (20)	14 (25.00)	0.34
Neck pain (*n* (%))	14 (6.48)	11 (6.87)	3 (5.36)	0.21
Claudication (*n* (%))	13 (6.02)	7 (4.38)	6 (10.71)	0.09
Imaging features				
Aortic regurgitation (*n* (%))	50 (23.15)	34 (21.25)	16 (28.57)	0.27
Pulmonary hypertension (*n* (%))	34 (15.74)	9 (5.63)	28 (50.00)	**< 0.001**
Type I	61 (28.24)	52 (32.5)	9 (16.07)	**0.001**
Type IIa	12 (5.56)	9 (5.63)	3 (5.36)	
Type IIb	25 (11.57)	**11 (6.88)**	**14 (25.00)**	
Type III	12 (5.56)	12 (7.50)	0 (0)	
Type VI	12 (5.56)	10 (6.25)	2 (3.57)	
Type V	94 (43.52)	66 (41.25)	28 (50)	
Laboratory results				
Haemoglobin (mean ± SD, g/L)	117.78 ± 20.18	117.70 ± 20.22	118.04 ± 20.24	0.92
White blood cells (mean ± SD, 10^9^/L)	9.29 ± 10.27	9.30 ± 11.05	9.24 ± 7.52	0.97
Platelets (mean ± SD, 10^9^/L)	287.33 ± 110.05	297.33 ± 111.67	256.94 ± 99.98	**0.02**
Erythrocyte sedimentation rate (mmHg)	37.25 ± 33.84	38.66 ± 33.66	33.11 ± 34.33	0.31
C-reactive protein (mg/L)	20.16 ± 31.31	21.40 ± 31.97	18.30 ± 29.41	0.54
Interleukin-6 (pg/L)	10.16 ± 14.19	10.17 ± 13.95	10.11 ± 15.06	0.98

*P value: comparison between patients with and without pulmonary artery involvement (PAI)

In contrast with patients without PAI, patients with PAI suffered from more chest pain/chest distress (24 (42.86%) vs. 27 (16.88%), *P* < 0.001), cough/sputum (6 (10.71%) vs. 3 (1.88%), *P* = 0.008), oral ulcer (6 (3.75%) vs. 6 (10.71%), *P* = 0.04), and PH (28 (50.00%) vs. 9 (5.63%), *P* < 0.001).

### PAI

**Fig. 1:**
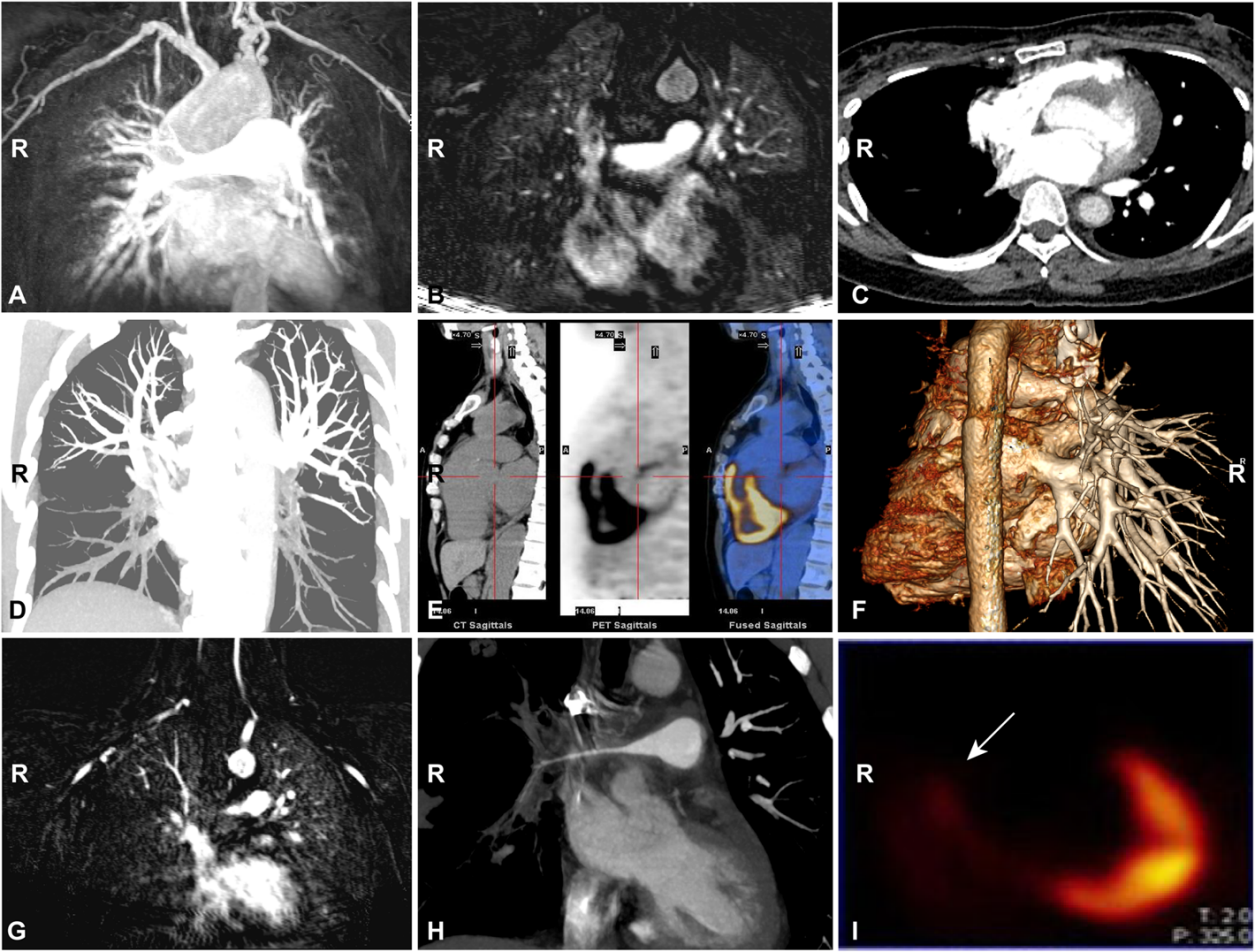
Imaging of PA lesions in TA patients. **a** Dilation of the PT upon CTA. **b** Thickness of the PT upon MRA. **c** Stenosis of the right main PA upon CTA. **d** Thrombosis of the lower PAs on both sides upon CTA. **e** Inflammation of the pulmonary trunk root upon PET-CT (SUV 4.1). **f** The absence of left PAs and stenosis of the right main PA (reconstructed image of CTA). Pulmonary MRA (**g**), CTA (**h**), and VQ scan (**i**) of a patient with TA. MRA shows a fine right main PA and low perfusion in the right lung (**g**). CTA demonstrates a fine right main PA and fewer PA branches in the right lung (**h**). Lung VQ scan showed filling defect of the complete right lung (**i**)

Among 56 patients with PAI, 14 (25%) patients presented PT, right pulmonary artery (RPA), and left pulmonary artery (LPA) lesions; 10 (17.86%) patients had RPA and LPA involvements; 7 (12.5%) and 3 (5.36%) patients demonstrated PT and RPA or LPA lesions, respectively, while single PT, RPA, or LPA involvement was observed in 10 (17.86%), 8 (14.29%), and 5 (8.93%) patients, respectively. Their specific features were listed in Table 2, and the representative images were shown in Fig. 1. Among these patients, 105 PAs were involved, including PT (34, 32.38%), RPA (38, 36.19%), and LPA (33, 31.43%). The most frequent presentation was vascular stenosis (36/105, 34.29%), followed by vascular dilation (25/105, 23.81%) and vascular enhancement (16/105, 15.24%). RPA or LPA presented predominantly as vascular stenosis (right, 22/38, 57.89%; left, 13/33, 39.39%), whereas the PT presented mainly as vascular dilation (22/34, 64.71%). Nine patients with low perfusion upon MRA underwent lung VQ scans (Fig. 1g, i; Supplementary Figure 2A, 3), which confirmed filling defects in multiple arteries, indicating that TA patients with PAI can also have small vessel lesions.

**Table 2:** Imaging features of involved pulmonary arteries

Imaging feature	PT (*n* = 34)	RPA (*n* = 38)					LPA (*n* = 33)				Total
		Main	U	M	L	Total	Main	U	L	Total	
Thickness (*n* (%))	1 (2.94)	1	2	0	0	3 (7.89)	0	1	1	2 (6.06)	6 (5.71)
Dilation (*n* (%))	**22 (64.71)**	1	0	0	0	1 (2.63)	2	0	0	2 (6.06)	**25 (23.81)**
Stenosis (*n* (%))	1 (2.94)	7	12	0	3	**22 (57.89)**	5	4	4	**13 (39.39)**	**36 (34.29)**
Occlusion (*n* (%))	0 (0)	5	0	0	1	6 (15.79)	5	0	1	6 (18.18)	12 (11.43)
Thrombosis (*n* (%))	0 (0)	**2**	0	1	0	3 (7.89)	**5**	0	2	7 (21.21)	10 (9.52)
Enhancement (*n* (%))	10 (29.41)	3	0	0	0	3 (7.89)	3	0	0	3 (9.09)	**16 (15.24)**
Total	34 (100)	19	14	1	4	38 (100)	20	5	8	33 (100)	105 (100)

*PT* pulmonary trunk, *RPA* right main pulmonary artery, *LPA* left main pulmonary artery, *U* upper pulmonary artery, *M* middle pulmonary artery, *L* lower pulmonary artery

### PH and cardiac function

Among 56 patients with PAI, half of them (28/56, 50%) had PH, which were graded as severe in 9 (16.07%), moderate in 10 (17.86%), and mild in 9 (16.07%) patients. Three patients performed RHC (Supplementary Table 1). Based on PH causes, 16 patients belonged to group 2 (due to left heart disease) and 7 patients pertained to group 4 (due to PAI), whereas 5 patients had the underlying causes of group 2 and group 4 (PAI as well as aortic or left heart insufficiency).

According to the NYHA classification, twenty-six patients (46.43%) showed advanced NYHA function (III, 20, 35.71%; IV, 6, 10.71%), implying that PAI reduced cardiac function to a great extent.

### Pulmonary parenchymal lesions in the regions corresponding to PAI

**Fig. 2:**
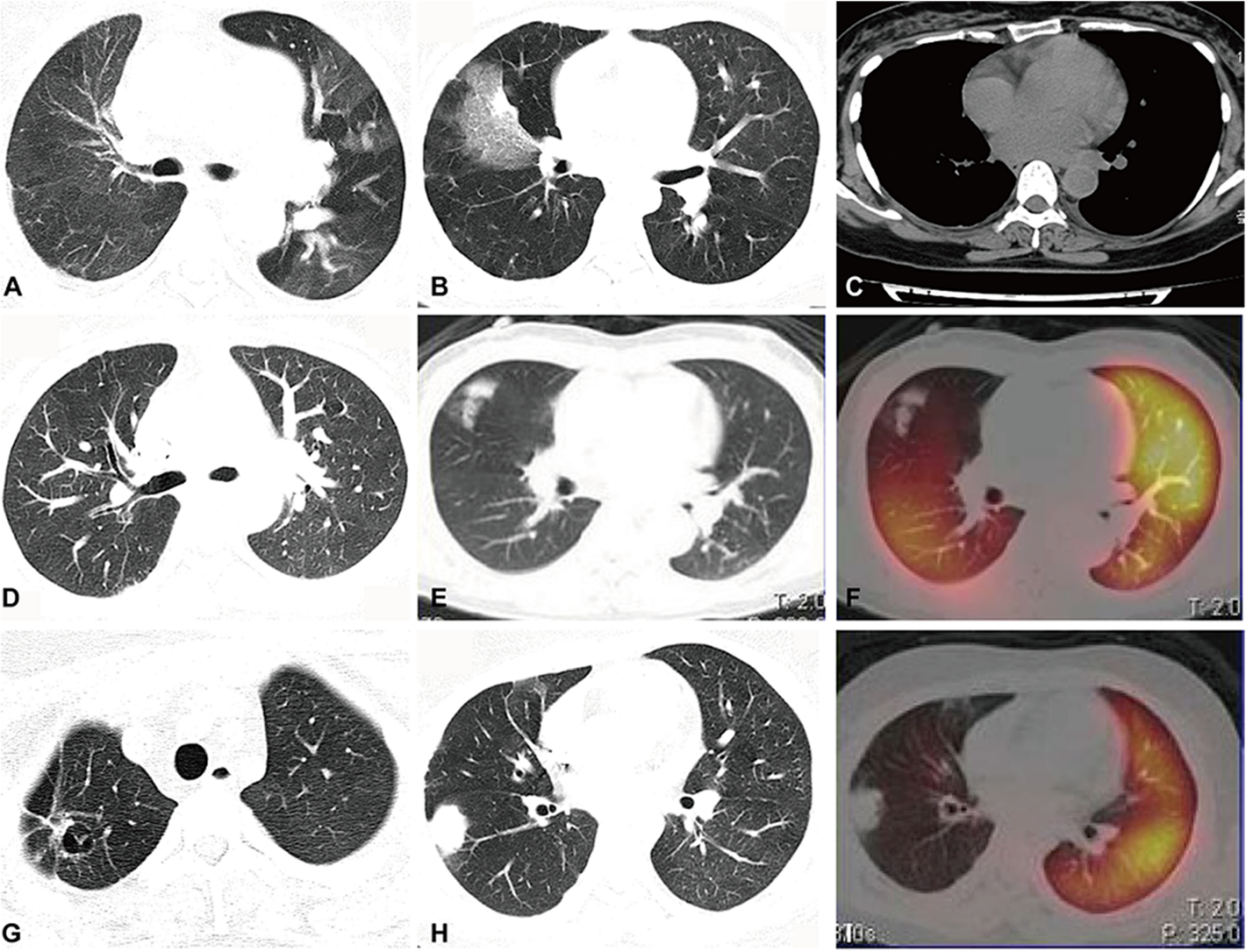
Pulmonary lesions on HRCT. **a** The mosaic sign in the left lung of a patient with stenosis of the left main PA. **b** Pulmonary infarction of the right middle lobe in a patient with severe stenosis of the right main PA. **c** Mild pleural effusion on the left side in a patient with pulmonary trunk dilation and pulmonary hypertension. **d** Bronchiectasis in the right lung in a patient with stenosis of the right main PA and its distant branches. Ground-glass opacity (**e**) in the right upper lobe of a TA patient with right upper pulmonary arterial branches involvement (**f**). Cavitation (**g**) and mass-like consolidation (**h**) in the patient with severe stenosis of the right main PA (**i**)

**Fig. 3:**
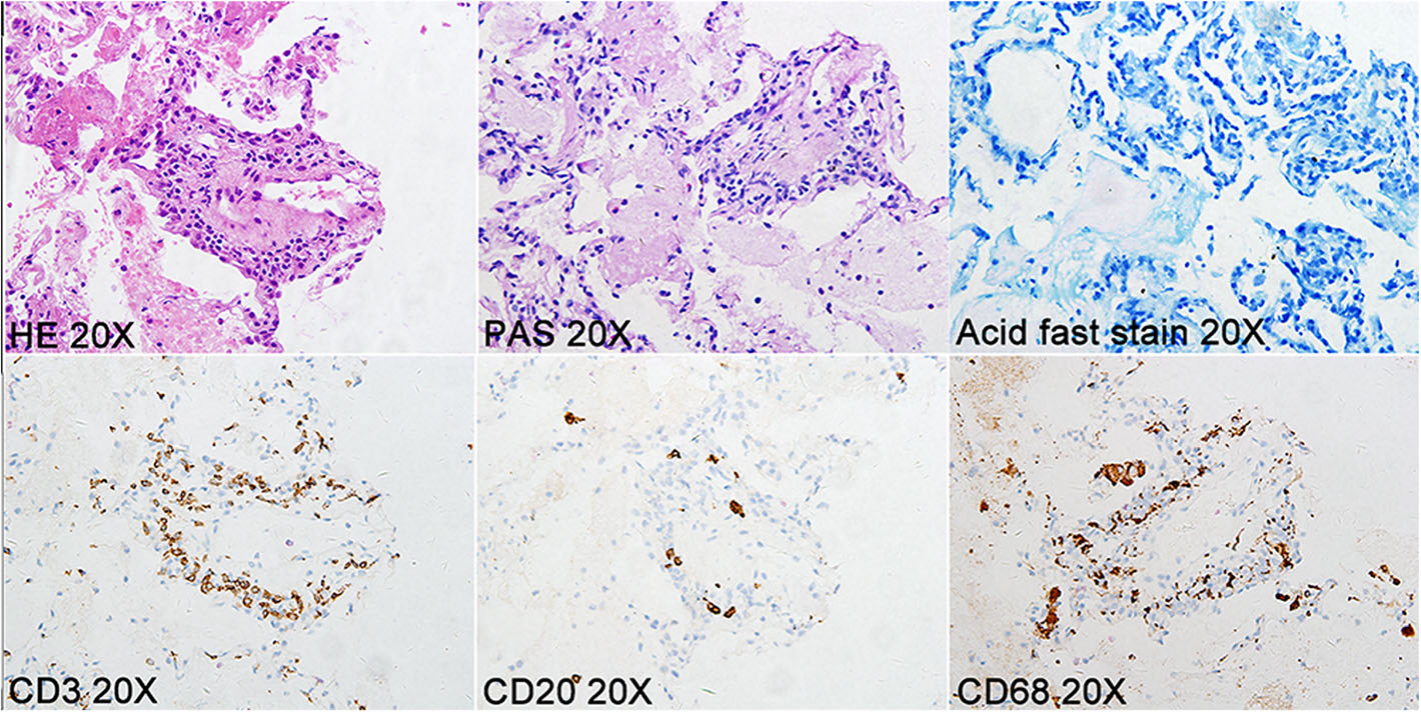
Pathological features of pulmonary parenchymal lesions. The pathological of pulmonary lesions indicated vasculitis of the pulmonary small vessels with CD3-positive cell and CD68-positive cell infiltration. Special staining (PAS and acid-fast stain) did not find evidence of fungal or tuberculosis bacillus infections

Low perfusion in PAs can decrease oxygen delivery to tissues and impair cell function, leading to tissue injury. Among 56 patients with PAI, 21 (37.50%) patients presented with abnormal pulmonary parenchymal lesions, including the mosaic sign in 7 patients, infarction in 6 patients, pleural effusion in 4 patients, ground-glass opacities in 3 patients, bronchiectasis in 2 patients, cavitation in 1 patient, and atelectasis in 1 patient. Representative images of low perfusion and corresponding pulmonary parenchymal lesions were shown in Fig. 2. Lung biopsy was carried out in two of the 56 patients with PAI, which indicated haemorrhagic infarction of pulmonary lesions, accompanied by fibrinoid necrosis of the small vessels and infiltration of inflammatory cells (Fig. 3). Special stainings such as PAS staining and acid-fast staining were negative, which excluded common pulmonary infections (Fig. 3). These results confirmed vasculitis in the pulmonary parenchymal lesions of TA patients.

### Follow-up of patients with PAI

Thirty-six patients completed ≥ 3-months follow-up, and the median duration of follow-up was 12 (interquartile range, 7–31.5) months. Among them, two (5.56%) patients were treated with only prednisone; 32 (88.89%) patients used prednisone combined with immunosuppressants (5 AZA, 8 CYC, 12 LEF, 1 MTX, 4 LEF, and sirolimus, 2 LEF and CYC), while the remaining 2 (5.56%) patients were treated with prednisone combined with biologics (1 tofacitinib, 1 tocilizumab). Besides, two patients also accepted surgical intervention (stent implantation and balloon dilation for each) due to bilateral PAI. At the final visit, 13/36 (36.11%) patients continued to have active disease or disease relapse, and 2/36 (5.26%) patients died due to pulmonary thrombosis combined with heart failure (duration of follow-up was 4 and 12 months, respectively). Both patients had PAI (dilated pulmonary trunk and fine left pulmonary arterial branches in one patient, left pulmonary artery stenosis in another patient) when they were diagnosed.

**Fig. 4:**
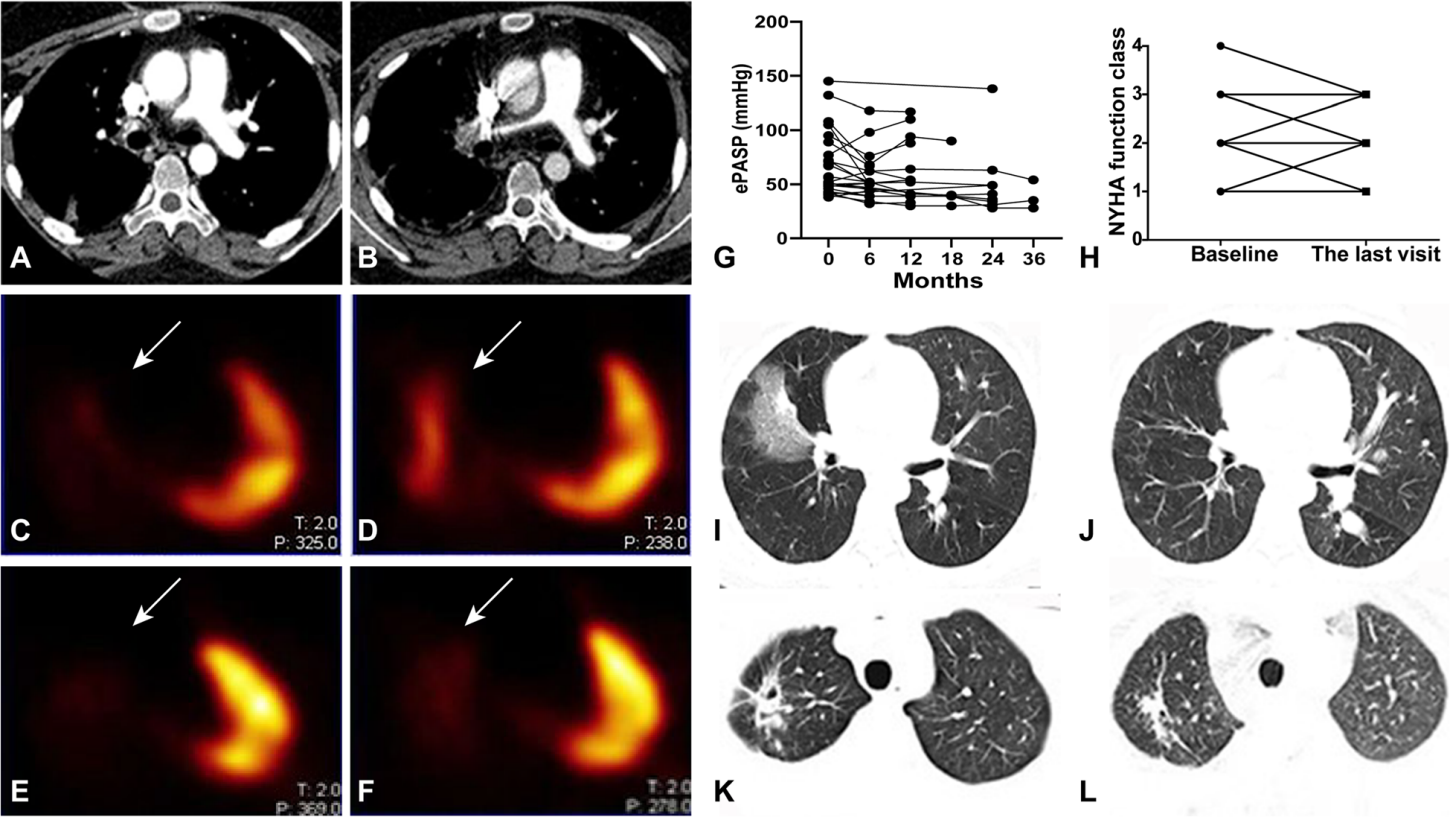
Pulmonary and cardiac conditions after treatment. **a**–**f** Pulmonary arterial changes before and after treatment: the right main pulmonary artery was shown upon CTA after balloon dilation intervention as well as immunosuppressive treatment in one patient (prednisone and azathioprine; **a** before treatment; **b** after treatment); blood supply to the right lung was also increased upon lung VQ scan after 1 year of treatment with prednisone and sirolimus in another patient (**c**, **e** before treatment; **d**, **f** after treatment). **g** The ePASP upon ultrasonography tended to decrease during patients’ follow-ups (baseline, 62.32 ± 28.76 mmHg, *n* = 27; 6th month, 58.73 ± 24.03 mmHg, *n* = 15; 12th month, 62.69 ± 29.75 mmHg, *n* = 13; 18th month, 49.75 ± 27.21 mmHg, *n* = 4; 24th month, 52.11 ± 34.01 mmHg, *n* = 9; 36th month, 39 ± 13.45 mmHg, *n* = 3) despite of treatment regimens. **h** Patients’ cardiac function was improved in most patients despite of treatment regimens. **i**, **j** Infarcted lesions dissipated after 12 months of treatment with prednisone and AZA as well as agents for PH in one patient. **k**, **l** The cavitation in the right lung apex became smaller after treatment with prednisone and sirolimus for 7 months in one patient

Among them, 31 (86.11%), 26 (72.22%), and 18 (50.00%) patients completed the 6th, 12th, and 24th month MRA, respectively. At the time point of 6 months, no obvious changes were observed except for the two patients with surgical interventions (stent implantation and balloon dilation for each); at the time point of 12th month, one (1/26, 3.85%) patient’s pulmonary arterial lesions got worsened, while the remaining (25/26, 96.15%) patients’ pulmonary arterial involvement kept stable; at the time point of 24th month, pulmonary arterial lesions were improved, stable, and more severe in 2 (11.11%), 14 (77.78%), and 2 (11.11%) patients, respectively. Representative pulmonary CTA and VQ scan images post-treatment were shown in Fig. 4a–f and Supplementary Figure 2B.

Although patients’ pulmonary arterial lesions did not change a lot after medical treatment, their overall conditions were improved. Cough/sputum in four cases after medications and haemoptysis in three cases (one after medication combined with endovascular treatment, two after medications) had resolved. In addition, ePASP upon ultrasonography tended to decrease during patients’ follow-ups (baseline, 62.32 ± 28.76 mmHg, *n* = 27; 6th month, 58.73 ± 24.03 mmHg, *n* = 15; 12th month, 62.69 ± 29.75 mmHg, *n* = 13; 18th month, 49.75 ± 27.21 mmHg, *n* = 4; 24th month, 52.11 ± 34.01 mmHg, *n* = 9; 36th month, 39 ± 13.45 mmHg, *n* = 3; Fig. 4g). Eleven patients showed improvement in cardiac function (from IV to III in three patients, from III to II in four cases, from II to I in four patients), 20 patients showed no change, and cardiac function worsened in three cases (from II to III in two cases, from I to II in one patient, Fig. 4h).

Nine patients with lung parenchymal lesions at baseline had repeated pulmonary CT (previously, four cases had pulmonary infarction, two had pleural effusion, two had the mosaic sign, and one had pulmonary cavitation). After treatment, pulmonary infarction in 2/4 (50%) patients had dissipated, 2/2 (100%) patients with pleural effusion had a reduced effusion volume, and 1/1 (100%) patient with pulmonary cavitation has improved. However, no changes were observed regarding other pulmonary lesions upon repeat pulmonary CT. Representative pulmonary CT images were demonstrated in Fig. 4i–l.

## Discussion

We discovered that one quarter of TA patients can have PAI, which presented in the PT, main PA, lobar artery, and segmental (or even sub-segmental) arteries. The prevalence of PH was ~ 15% in patients with TA whereas, among TA patients with PAI, it was 50%, which contributed to the impairment of cardiac function. PAI could also cause pulmonary parenchymal lesions (37.5%). Therefore, PAI in patients with TA can affect multiple aspects of cardiopulmonary functions, which worsens the prognosis.

One quarter of patients had PAI, a figure similar to that reported in other countries (5 to 36.7%) [9, 25]. Not all patients presented with symptoms such as cough or haemoptysis, which suggested that PAI in TA can be silent [26]. In addition, we did not find an association between PAI and disease activity. This finding was in accordance with a report from China [27] but contrary to a study from Korea that discovered PAI in a high proportion of patients with clinically active TA [5]. Given that TA is indolent, patients might be diagnosed at different stages of TA. Hence, the relationship between PAI and disease activity might differ in different studies.

Stenosis was the most common imaging presentation of the involved PAs, followed by dilation and emboli. Two studies have shown that stenosis or occlusion was the most common presentation of involved PAs in TA [25, 28]. Additional lung VQ scans for patients with low perfusion upon MRA revealed pulmonary emboli to be very common in TA patients, which involved multiple segmental (or sub-segmental) PAs. Therefore, low perfusion upon MRA should not be ignored in patients with TA, and pulmonary VQ scans are valuable for the detection of emboli in the small arteries.

PH is a direct result of PAI. In the present study, half of the patients with PAI suffered from PH. Among them, the ratio of left heart disease-related PH (group 2) was more than that of pulmonary embolism-related PH (group 4), a finding that is in accordance with a report from Wang and colleagues [28]. This phenomenon can be explained by the higher prevalence of aortic involvement than PAI in TA. Moreover, we observed that the PH and cardiac insufficiency were moderate to severe in patients with PAI, implying cardiopulmonary function was badly damaged in patients with PAI.

In TA, PAI can also induce pulmonary parenchymal lesions, which was consistent with the past reports [29, 30]. Various pulmonary lesions (the mosaic sign, infarction, pleural effusion, bronchiectasis, cavitation) were observed in regions corresponding to PAI. The mosaic sign and pulmonary infarction are direct pulmonary manifestations on account of hypoperfusion [11, 31, 32]. Recurrence of pleural effusions has been reported to be associated with disease activity [33, 34]. Bilateral effusions are probably induced by cardiac insufficiency, whereas a unilateral effusion might be related to pleural inflammation or small vessel vasculitis in peripheral pulmonary areas [35]. Bronchiectasis has been reported in one case study of TA [36], which we assumed was the result of the involvement of the bronchial arteries in TA. Poor blood perfusion can cause chronic destruction of bronchial tissue and further bronchiectasis. Moreover, cavitation has been reported in TA [37, 38], which can be treated with glucocorticoids combined with immunosuppressants, further confirming that cavitation is mediated by abnormal autoimmune reactions.

Patients with PAI should be monitored closely and treated comprehensively. In the present study, pulmonary thrombosis developed in two patients with primary PAI, which was probably due to endothelial injury under the condition of vascular inflammation. Since they also had chronic heart failure due to long-term PH, it was assumed that their cardiopulmonary dysfunction contributed to their death to a great extent. Besides the usual treatment for TA, PH-targeting agents are necessary for patients with PH (especially group 4 PH). In some cases, surgery is also indicated. Our study demonstrated that symptoms, pulmonary pressure, and cardiac function were improved markedly after treatment in most patients, but some patients did not respond well. Hence, ascertaining the factors responsible for such different therapeutic responses must be undertaken. In addition, novel treatment strategies must be explored. These strategies will lay the foundation for future precise treatment for TA.

Some limitations should be addressed in the present study. Firstly, since RHC is invasive and would be contradicted in some patients with vascular stenosis, ePASP upon echocardiography was mainly applied in the present study to evaluate patients’ PH, which may decrease the accuracy to some extent. Secondly, pulmonary arterial thrombosis might be low estimated since pulmonary CTA and VQ scan were not routinely performed for all the patients enrolled.

## Conclusions

PAI is common in TA patients. Physicians should be alerted to this even if obvious pulmonary symptoms are absent because it can cause PH, cardiac insufficiency, and pulmonary parenchymal lesions, which will worsen the prognosis.

## Supplementary information

**Additional file 1: Figure S1.** The flow chart of this study. **Table S1.** mPAP detected by RHC. **Figure S2.** Improvement of perfusion defects upon lung VQ scan in a patient with TA. **Figure S3.** Low perfusion upon MRA in patients with TA.

## Data Availability

The dataset supporting the conclusions of this article is included within the article and its additional file.

## Abbreviations

TA: Takayasu arteritis
PH: Pulmonary hypertension
DSA: Digital subtraction angiography
MRA: Magnetic resonance angiography
CTA: Computed tomography angiography
PET-CT: Positron emission tomography-computed tomography
VQ scan: Ventilation/perfusion scan
HRCT: High-resolution computed tomography
ESR: Erythrocyte sedimentation rate
CRP: C-reactive protein
CYC: Cyclophosphamide
MTX: Methotrexate
AZA: Azathioprine
LEF: Leflunomide
SUV: Standard uptake value
RHC: Right heart catheterization
PASP: Pulmonary arterial systolic pressure
NYHA: New York Heart Association
PT: Pulmonary trunk
RPA: Right pulmonary arteries
LPA: Left pulmonary arteries
