# Supplementary material for: Involvement of the pulmonary arteries in patients with Takayasu arteritis: a prospective study from a single centre in China
Source: Arthritis Res Ther. 2020 Jun 5;22:131. doi: 10.1186/s13075-020-02203-1 (PMC7275430; doi:10.1186/s13075-020-02203-1)
Supplement: Supplementary file 1 — Additional file 1: Figure S1. The flow chart of this study. Table S1. mPAP detected by RHC. Figure S2. Improvement of perfusion defects upon lung VQ scan in a patient with TA. Figure S3. Low perfusion upon MRA in patients with TA. [file 13075_2020_2203_MOESM1_ESM.doc]

**Supplementary Figure 1. The flow chart of this study**

**
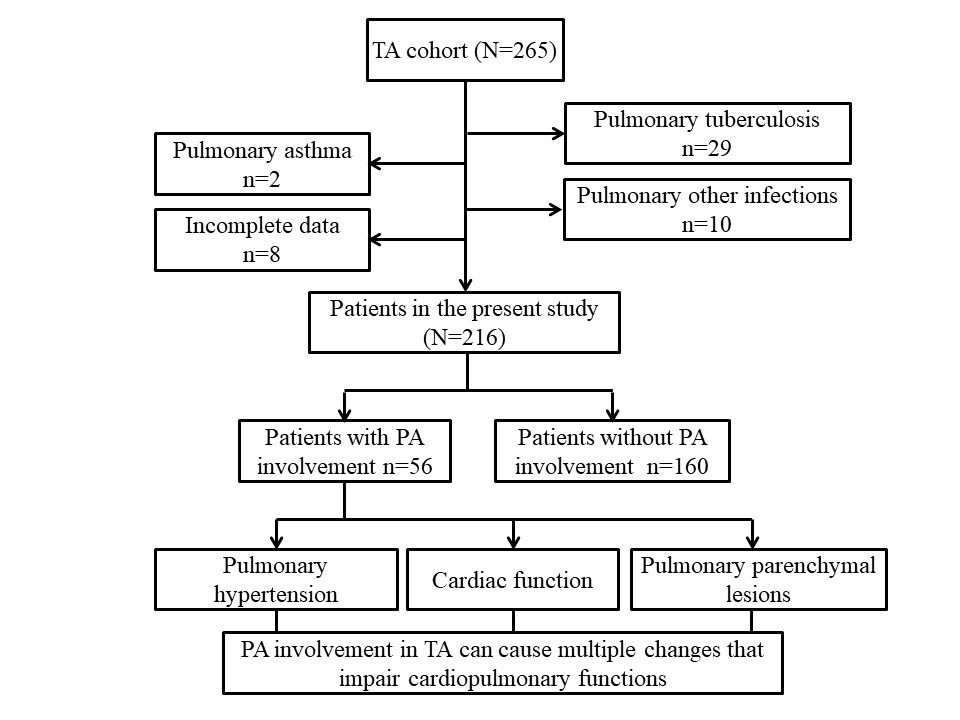
**

**Supplementary Table 1. mPAP detected by RHC**

| **mPAP** | **1^st^ patient** | **2^nd^ patient** | **3^rd^ patient** |
| --- | --- | --- | --- |
| **Baseline (mmHg)** | 54 | 66 | 88 |
| **1^st^ year post treatment (mmHg)** | **/** | 49 | **/** |

**Supplementary Figure 2. Improvement of perfusion defects upon lung VQ scan in a patient with TA**

**
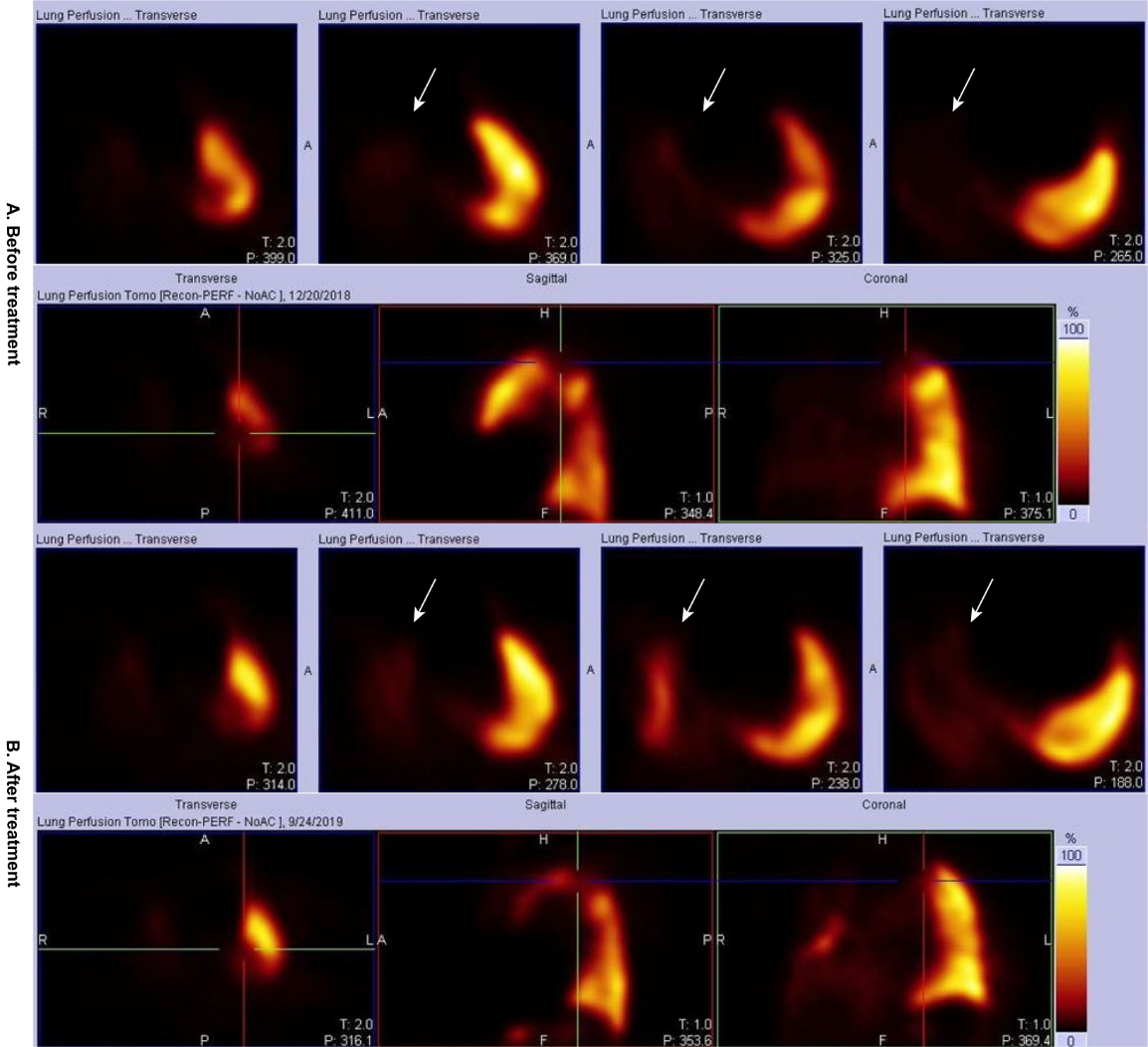
**

1. Filling defects of the complete right lung and left apicoposterius segmentum were demonstrated by lung VQ scan in a patient with TA before treatment;
2. The filling defects of the right lung and left apicoposterius segmentum were improved after 1 year treatment with prednisone and sirolimus .

**Supplementary Figure 3. Low perfusion upon MRA in patients with TA**

**
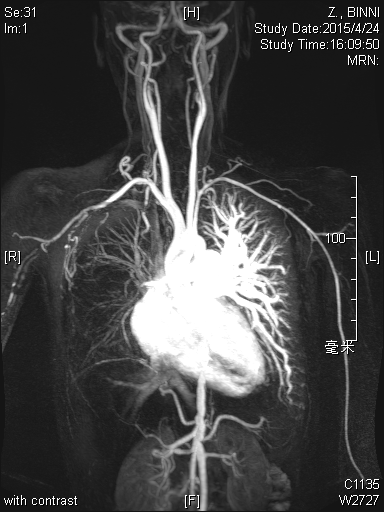

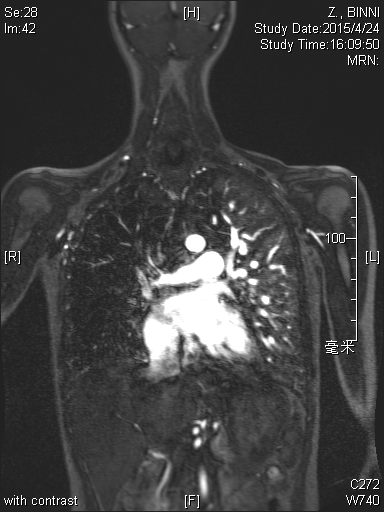
**

**A**

**B**

**
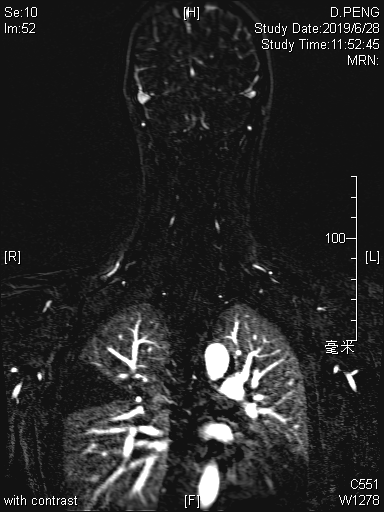

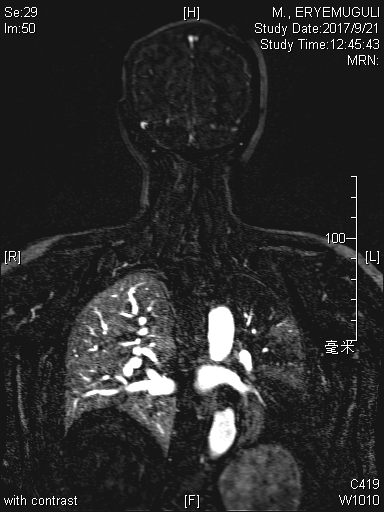
**

**D**

**C**

Panel A and B are from one person, while panel C and D are from two different persons. Panel A demonstrated severe stenosis of right pulmonary arteries, therefore, low perfusion of the right lung compared with the left lung can be seen in its coronal scan (panel B). Similarly, when there was stenosis of other pulmonary arteries, the perfusion of the corresponding area was low such as lower left part of the patient in panel C and left lung of the patient in panel D
